# Gut Microbiota and Ageing: A Critical Crosstalk in Alcohol-Related Liver Disease

**DOI:** 10.3390/microorganisms14071469

**Published:** 2026-07-03

**Authors:** Yupin Tan, Yirui Hu, Zhuang Cao, Xinyang Wang, Yonggang Yuan, Huikuan Chu

**Affiliations:** Division of Gastroenterology, Union Hospital, Tongji Medical College, Huazhong University of Science and Technology, 1277 Jiefang Avenue, Wuhan 430022, China; u202210276@hust.edu.cn (Y.T.); 15550884611@163.com (X.W.); godblessyyg@gmail.com (Y.Y.)

**Keywords:** alcohol-related liver disease, gut-liver axis, dysbiosis

## Abstract

Alcohol-related liver disease (ALD) poses a significant global health burden, driven by complex mechanisms including oxidative stress, inflammation, and gut–liver axis disruption. While the individual roles of gut microbiota dysbiosis and ageing in ALD pathogenesis are increasingly recognized, their synergistic interaction remains poorly understood. This review synthesizes current evidence to argue that there is an interaction between ageing and the gut microbiota that collectively amplifies progression of ALD. Specifically, ageing promotes gut dysbiosis through immunosenescence (e.g., reduced IgA diversification and antimicrobial peptide decline), intestinal barrier failure, and altered microbial metabolite profiles (e.g., decreased short-chain fatty acids and dysregulated bile acid metabolism). Conversely, dysbiosis-derived metabolites and endotoxins modulate ageing-related signaling pathways, including SIRT1, FOXO, and Nrf2, thereby accelerating hepatic cellular senescence, inflammation, and fibrogenesis. Furthermore, we also discussed the typical microbial changes in ALD. These include an increase in the Proteobacteria, a decrease in the Bacteroidetes, as well as imbalances in fungi and viruses. In ageing, similar but distinct shifts occur, such as reduced microbial diversity, decreased short-chain fatty acid producers, and increased intestinal permeability. Therapeutic strategies targeting the gut microbiota (probiotics, fecal microbiota transplantation) or ageing-related pathways (SIRT1 activators) hold promise. Future research priorities include validating ageing-associated microbial signatures as predictors of ALD progression and testing microbiota-targeted interventions in aged preclinical models. Collectively, this review identifies the microbiota–ageing axis as a tractable therapeutic target for ALD and provides a framework for future mechanistic and translational studies.

## 1. Introduction

Alcohol-related liver disease (ALD) represents a spectrum of hepatic injury induced by excessive alcohol consumption, progressing from simple steatosis and alcoholic hepatitis to advanced fibrosis, cirrhosis, and ultimately liver failure [1,2]. ALD causes approximately 3.3 million deaths annually, accounting for 5.9% of global mortality [3]. Alcohol consumption is the leading cause of cirrhosis worldwide, increasing the risk of liver disease-related death by 260-fold [4,5], posing a significant global health challenge. Mechanistically, alcohol is primarily metabolized in hepatocytes by alcohol dehydrogenase (ADH) and aldehyde dehydrogenase (ALDH) to generate acetaldehyde and acetate, with the microsomal ethanol-oxidizing system (CYP2E1) becoming increasingly involved at higher intake levels. This process generates reactive oxygen species and oxidative stress that directly injure hepatocytes [6,7]. In the intestinal tract, alcohol and its metabolites disrupt epithelial tight junctions, increase mucosal permeability, and alter gastric acid secretion, thereby promoting bacterial overgrowth and translocation, which initiate and perpetuate gut dysbiosis in ALD [8,9]. Current management relies heavily on sustained alcohol abstinence, yet achieving this remains difficult for many patients. Furthermore, treatment options are limited, with no universally approved pharmacological therapies specifically for ALD, making liver transplantation the key intervention for end-stage disease [10]. This pressing clinical reality compels us to investigate its underlying mechanisms.

Various mechanisms contribute to the development of ALD, including the direct toxicity of alcohol metabolites, oxidative stress, pro-inflammatory factors (such as TNF-α), autophagy disorders, and even gender, which has been confirmed as a risk factor [11]. Recent studies have shown significant alterations in the gut microbiota composition of ALD patients compared to healthy individuals, with notable differences even among patients at different stages of ALD. These gut microbiota changes, including an increase in Proteobacteria and a decrease in Bacteroidetes, along with intestinal barrier disruption, contribute to disease progression [12,13,14,15]. Targeting these microbiota may provide a novel therapeutic approach for ALD.

Moreover, ageing contributes to the development of ALD. Middle-aged and elderly mice are more susceptible to alcohol-induced liver damage, inflammation, and oxidative stress compared to younger mice, leading to more severe ALD [16]. For instance, recent studies have shown that as people age, the probability of Gram-negative bacteria invading the ileum increases, leading to excessive proliferation of B cells in the intestinal germinal center (GC) of the ileum, and causing cellular senescence due to the accumulation of DNA damage. The senescent GC B cells reduce the production and abundance of IgA, thereby affecting the composition of the intestinal microbiota [17]. Interestingly, ageing is also associated with changes in the gut microbiota, manifested as reduced species diversity and increased intestinal permeability. Notably, specific microbial alterations observed in the elderly, such as shifts in Proteobacteria and Bacteroidetes, are similarly observed in patients with ALD [18]. This indicates that age-related microbial changes are associated with the pathogenic mechanisms of ALD.

Beyond compositional shifts, gut microbiota metabolites, such as bile acids, short-chain fatty acids, and endotoxins, regulate ageing and are closely associated with ALD development. At the cellular level, the ageing process drives the accumulation of senescent cells within the liver, which acquires a distinct pro-inflammatory and tissue-damaging secretory phenotype; this cellular senescence exacerbates chronic liver injury, fuels inflammation and fibrosis, and creates a microenvironment conducive to disease progression, including hepatocellular carcinoma [19]. Furthermore, signaling pathways related to both ageing and ALD, such as those involving sirtuin 1 (SIRT1), forkhead box, class O (FOXO) and nuclear factor erythroid-derived 2-like 2 (Nrf2), play crucial roles in disease progression [20]. The gut microbiota may influence the progression of ALD by modulating ageing processes. Moreover, ageing-related genes alter the intestinal flora, influencing the progression of ALD. Interactions between the gut microbiota and ageing represent a promising therapeutic target for ALD.

Currently, dysbiosis of the gut microbiota promotes the progression of ALD, while ageing also contributes to ALD-related liver damage by exacerbating liver inflammation and oxidative stress. Furthermore, certain microbial metabolites regulate ageing, and ageing has been shown to alter the composition of the gut microbiota and impair intestinal barrier function. However, it remains unclear whether and through what specific mechanisms the interaction between the gut microbiota and ageing synergistically accelerates the development of ALD, rather than each acting independently. To address this gap, this article first summarizes the compositional and functional changes in gut bacteria, fungi, and viruses in the context of ALD, and then analyzes how ageing influences the pathogenesis of ALD at the cellular and signaling pathway levels. Finally, we explore the specific mechanisms underlying the mutual influence and regulation between the gut microbiota and ageing, emphasizing the impact of this interaction on the progression of ALD. In summary, there is an interaction between the gut microbiota and ageing, and it is highly likely that the two act synergistically to exacerbate liver damage in ALD. Therefore, this review aims to provide a theoretical basis for the precise prevention and treatment of ALD in elderly patients and to lay the foundation for the development of targeted intervention strategies.

## 2. The Gut Microbiota and ALD

Alcohol intake, influenced by environmental factors, diseases, and other triggers, leads to shifts in the gut microbiota, affecting both its composition and function. Regarding composition, the mucosa-associated colonic microbiome in patients with alcohol use disorders (both with and without liver disease) compared to healthy individuals reveals an imbalance (Table 1). A decrease in the median abundances of Bacteroidetes and Lactobacillus, alongside an increase in Proteobacteria and Fusobacteria, have been observed and may be linked to endotoxemia [21,22]. Functional changes directly affect the host’s immune system and can be investigated through metabolomics, proteomics, and transcriptomics [23]. Furthermore, metabolic by-products of the gut microbiota, particularly bile acids, undergo significant alterations in ALD patients compared to healthy individuals. Additionally, changes in specific microbial strains may indicate the progression of ALD, offering insights into its diagnosis and prognosis [24]. In this context, probiotic supplementation emerges as a promising therapeutic strategy by directly countering these alcohol-induced dysbiotic changes. It has been demonstrated to restore microbial balance, fortify the intestinal barrier, and consequently reduce the translocation of bacterial products such as lipopolysaccharides, thereby ameliorating hepatic inflammation and injury in ALD [25,26].

### 2.1. Change in Gut Microbiota in ALD

#### 2.1.1. Bacteria

The overall change in intestinal bacteria in ALD is manifested as a decrease in diversity, a reduction in beneficial bacteria, and an increase in harmful bacteria [34]. At the phylum level, the abundance of Verrucomicrobiota, Proteobacteria, Actinobacteria, and Fusobacteria increases in ALD patients, while Bacteroidetes decreases [15,35,36]. However, discrepancies exist in various reports about changes in the Firmicutes phylum [35,36,37]. At the species level, the abundance of *Bifidobacterium*, *Lactobacillus*, and *Enterococcus* in the alcohol group is significantly lower than in the healthy group [38]. Enterobacteriaceae abundance in the feces of alcohol-related cirrhosis patients is 27 times higher than in healthy volunteers, making it the most common translocating bacterium in cirrhosis patients [39].

#### 2.1.2. Fungi

Similar to bacteria, changes in the fungal community manifest as reduced diversity and overgrowth of certain fungi, particularly *Candida albicans* [40,41]. At the phylum level, ALD patients exhibited significantly increased relative abundance of the Ascomycota and reduced abundance of the Basidiomycota phylum compared to healthy controls [42,43]. At the genus level, there was an increase in the abundances of *Candida* and *Debaryomyces*, and a decrease in the abundance of the beneficial genus *Saccharomyces* [32,42]. At the species level, increased abundance of *Candida albicans* is a common feature of fungal dysbiosis in ALD [40,44]. In contrast, the beneficial yeast *Saccharomyces cerevisiae* is often found to be depleted in ALD patients [32].

#### 2.1.3. Virus

A recent investigation revealed heightened viral diversity in fecal samples from individuals with ALD, particularly among those diagnosed with alcoholic hepatitis (AH). In these patients, phages targeting *Escherichia*, *Enterobacteria*, and *Enterococcus* were more prevalent, alongside notable rises in mammalian viral families such as *Parvoviridae* and *Herpesviridae* [33].

In summary, a marked alteration in the composition of the gut microbiota is a characteristic feature of ALD.

### 2.2. The Role of Gut Microbiota in the Diagnosis and Progression of ALD

The gut microbiota plays a pivotal role in the progression of ALD, with specific microbial characteristics demonstrating potential for promoting disease progression, diagnosis, and differential diagnosis. For instance, oral cytolysin-positive *Enterococcus faecalis* exacerbate ethanol-induced liver injury in mice, indicating the bacterium’s involvement in disease pathogenesis [45]. Moreover, cytolysin is associated with mortality in AH [24]. Research shows that severe AH exhibits increased fecal abundance of *Bacilli*, *Lactobacillales*, and *Veillonella* compared to healthy controls, alongside decreased abundance of *Eubacterium_g23*, *Oscillibacter*, and *Clostridiales*. This suggests that the microbiota can distinguish disease severity [46,47]. Furthermore, the increased abundance of *Bacteroides* and *Prevotella* in ALD patients compared to heavy drinkers without liver disease highlights their potential role in distinguishing ALD from alcohol use disorder [14]. While microbial characteristics can distinguish alcohol users from AH patients, they may not predict the severity of moderate to severe disease in AH [15]. In addition to bacterial composition, bacterial metabolites such as endotoxins and short-chain fatty acids have also been implicated in disease progression and may serve as complementary diagnostic tools [48].

Fungi such as *Candida albicans*, *Saccharomyces cerevisiae* and *Malassezia restricta* are also involved in the progression of ALD and promote alcoholic liver injury. Especially *Candida albicans*, its exotoxin candidolysin directly damages hepatocytes without altering intestinal barrier function. This is associated with disease severity and mortality in AH patients [31]. Similarly, specific immune responses against fungi, such as serum anti-saccharomyces cerevisiae antibody (ASCA), are relatively higher in AH patients and are also associated with an increased mortality rate in AH patients [32]. The *Malassezia restricta* drives liver inflammation by activating the Dectin-2 immune pathway, which is associated with an increased severity of ALD [49]. Some fungi also have potential for diagnostic differentiation. For example, *Candida*, *Malassezia* and *Scopulariopsis* are usually increased in ALD, while *Kazachstania* and *Mucor* are significantly increased in NAFLD [50].

As previously mentioned, *Enterococcus faecalis* is significantly increased in patients with AH, and the presence of cytolysin-positive *Enterococcus faecalis* correlates with the severity of liver disease and mortality. 89% of cytolysin-positive AH patients died within 180 days of admission, compared to only 3.8% of cytolysin-negative patients. Furthermore, mice receiving FMT from cytolysin-positive patients exhibited more severe ethanol-induced liver injury, steatosis, inflammation, and fibrosis than those receiving transplants from cytolysin-negative patients [51]. Furthermore, metagenomic analysis revealed a unique enrichment of *Herpesviridae* sequences in AH patients. This enrichment level was also significantly correlated with clinical disease severity and mortality risk [33].

Collectively, these findings underscore the bacteria, fungi, and viruses as key players in ALD pathogenesis, offering promising avenues for diagnostic biomarkers and targeted therapeutic interventions. However, the composition of the microbiota alone cannot fully explain the variations in ALD severity, particularly the increased susceptibility observed in the elderly population [16,52]. This suggests that the host’s ageing state may exacerbate the effects of liver injury by altering the gut microbiota [53]. As detailed below, many of the microbial changes observed in ALD (e.g., reduced Bacteroidetes, increased Proteobacteria, decreased SCFA producers) strikingly parallel those reported in the ageing gut, suggesting that ageing may prime the microbiota toward a pro-inflammatory configuration that amplifies alcohol-induced injury. Therefore, understanding how ageing interacts with the gut microbiota is crucial. In the following sections, we will focus on how ageing influences the pathogenesis of ALD at the cellular and molecular levels, laying the groundwork for subsequent discussions on the interactions between the two.

## 3. Changes Associated with Ageing in ALD

Recent studies have revealed the progression of ALD is closely intertwined with the ageing process. Physiological ageing increases susceptibility to ALD by impairing hepatic repair mechanisms, while ALD-driven pathological senescence accelerates liver function decline through oxidative stress, telomere shortening, and epigenetic dysregulation. In addition, ethanol metabolism produces acetaldehyde and ROS, directly causing DNA damage and telomere shortening, and activating the p53/p21 and p16-INK4a-Rb ageing pathways in liver cells [54,55]. At the same time, ethanol intake depletes the NAD^+^ reserves in the liver, reduces the deacetylation effects mediated by SIRT1 on FOXO and Nrf2, weakens the antioxidant defense capacity, and exacerbates ageing caused by oxidative stress [16,56,57,58]. This interplay occurs at multiple levels, from cellular senescence to ageing-related gene expression. Complementing this view, single-nucleus multiomic profiling has revealed that dysregulated RNA splicing in ALD prevents hepatocytes from transitioning to a proliferative progenitor-like state, trapping them in an unproductive intermediate state that may underlie regeneration failure and contribute to progressive cellular dysfunction [59].

### 3.1. Cellular Senescence and Inflammation

Cellular senescence in ALD is induced through telomere shortening (replicative senescence) and oxidative stress or acetaldehyde damage (stress-induced senescence). A key driver of pathology is the senescence-associated secretory phenotype (SASP), through which senescent cells release pro-inflammatory factors. SASP factors IL-8 and IL-17 activate Kupffer cells, recruit neutrophils infiltrating the liver, and promote the formation of neutrophil extracellular traps (NETs), which release abundant ROS leading to hepatocyte lipid peroxidation [60].

The role of neutrophils in ageing-associated ALD is complex. The conventional view holds that neutrophils are short-lived and prone to death, with their subsequent clearance by macrophages via “efferocytosis” aiding in maintaining tissue homeostasis and exerting anti-inflammatory effects [61]. However, in ALD, chronic alcohol exposure delays neutrophil apoptosis and primes them for NET formation [62,63]. NETs release abundant ROS and proteases that inflict lipid peroxidation upon hepatocytes, inducing secondary senescence in previously non-senescent cells and establishing a self-perpetuating cycle [64]. Thus, neutrophils in ageing-associated ALD do not merely undergo senescence themselves; rather, their dysfunctional persistence and NET release actively drive senescence in hepatocytes and exacerbate liver injury.

A recent longitudinal transcriptomic analysis of paired liver biopsies from AH patients confirmed that senescence-associated gene signatures are progressively upregulated during ALD progression and partially reversed upon disease resolution, further supporting a pathogenic role for senescence in AH [65]. Beyond transcriptional signatures, hepatocellular senescence has been linked to elevated circulating levels of SASP factors, particularly growth differentiation factor 15, which correlates with disease severity, corticosteroid response, and 90-day mortality in AH patients [66].

### 3.2. Key Ageing-Related Genes

Ageing-related genes involved in DNA repair, cell cycle regulation, and metabolism are critically involved in ALD pathogenesis. Their altered expression often has both beneficial and detrimental effects. Ageing genes such as SIRT1, the FOXO family, and Nrf2 play crucial roles in maintaining the liver’s antioxidative defenses, metabolic balance, and cell survival. When the expression of these genes is diminished or their function is impaired, the liver’s ability to defend against alcohol-induced damage may be weakened, accelerating the development of ALD.

The sirtuin family is involved in energy metabolism, DNA repair, and antioxidative defense through their deacetylase activity. Researchers have found that SIRT1/miR-223 expression exhibits an age-dependent decline, and the acetylation level of C/EBPα in elderly individuals is elevated by 2.5-fold compared to younger individuals. This heightened acetylation directly suppresses miR-223 transcription, thereby accelerating ALD progression [52]. Restoring SIRT1 improved liver morphology and reduced fibrosis in these models, highlighting its therapeutic potential [16]. Additionally, Nicotinamide Riboside (NR) reduces oxidative stress and improves mitochondrial function in ALD through the SIRT1-PGC-1α axis [67]. Thus, activating SIRT1 through NR supplementation may be a promising therapeutic strategy for ALD management.

Meanwhile, FOXO transcription factors regulate cell survival and genomic stability by controlling genes involved in apoptosis, cell cycle arrest, and DNA repair [68,69]. Alcohol exposure suppresses FOXO1 expression, which is regulated by the transcription factor MIR148A and significantly reduced in human AH samples [70]. The functional loss or reduced expression of FOXO genes may lead to the downregulation of liver antioxidative enzymes, such as superoxide dismutase (SOD) and catalase (CAT), thereby diminishing the liver’s capacity to clear ROS and promoting the production of pro-inflammatory cytokines in 3T3L1 adipocytes, further exacerbating the progression of ALD [57].

Nrf2 is a key antioxidative stress transcription factor that protects cells from oxidative damage by upregulating the expression of various antioxidative and detoxifying enzymes. In the context of 4.2% ethanol feeding, liver-specific Nrf2 knockout mice exhibited more severe liver damage compared to Nrf2 floxed control mice [71]. Bardag-Gorce et al. found that Nrf2 levels were significantly reduced in the liver of an ALD rat model, potentially compromising the liver’s antioxidative defense mechanisms and making cells more susceptible to the toxic effects of alcohol metabolites such as acetaldehyde [72]. The reduction in Nrf2 activity may also affect the liver’s detoxification capacity against other harmful substances, exacerbating liver damage and fibrosis. Moreover, studies have found that reversing ethanol-induced Nrf2 downregulation has potential protective effects against alcohol-related liver damage in mice [73].

Increased expression of ageing genes plays a key role in ALD development by promoting inflammation, oxidative stress, and fibrogenesis, thereby exacerbating liver damage. Conversely, the reduced expression or functional loss of certain ageing genes may also exacerbate ALD. Therefore, disease intervention strategies targeting these genes may offer new directions for the treatment of ALD. Crucially, these age-related changes in the host do not occur in isolation, but rather interact with and are further exacerbated by the alterations in the gut microbiota described earlier. For example, age-induced impairment of the intestinal barrier function facilitates the transmembrane transfer of microbial metabolites, whilst age-related suppression of the SIRT1/FOXO/Nrf2 signalling pathway renders hepatocytes more susceptible to damage from microbe-derived endotoxins. This mutually reinforcing interaction between host ageing and gut dysbiosis constitutes the “microbiota–ageing axis”, which we shall explore in detail at the mechanistic level in Section 4.

## 4. Gut Microbiota and Ageing

Having discussed the microbial changes in ALD and the age-related alterations that increase host susceptibility separately, we now examine the bidirectional interactions between them. As mentioned earlier, the gut microbiota and host ageing do not act independently; rather, they mutually promote the progression of ALD. This section focuses on three related mechanisms, intestinal barrier integrity, immune inflammation and the production of microbial metabolites, to illustrate how the interaction of these factors exacerbates liver damage.

### 4.1. Intestinal Barrier Integrity in the Microbiome–Ageing Axis

Indeed, alterations in microbiota lead to compromised intestinal barriers and increased gut permeability [74]. Dysbiosis in the microbiome can precede and predict age-related intestinal barrier failure [75]. Gut bacteria or bacterial products such as PAMPs and MAMPs leak into the bloodstream, disseminating to other areas. This induces inflammation characterized by the production of reactive oxygen species, TNF, IL-1β, IL-18, and IL-6 [76]. This inflammation stimulates a systemic immune response in the host, resulting in a chronic pro-inflammatory state. The decline in protective bacteria such as *Clostridium* and *Bifidobacterium*, essential for maintaining intestinal barrier integrity, increases the risk of gut leakage and systemic endotoxemia [77]. Such changes could potentially have detrimental implications for healthy ageing and longevity.

Ageing promotes disruption of epithelial barrier integrity, translocation of bacterial products, and elevation of serum pro-inflammatory cytokine levels. For example, intestinal fatty acid binding protein (I-FABP) is a key indicator of microbial-related epithelial cell damage and destruction, as well as a biomarker of intestinal permeability. Compared to young mice receiving PBS, young mice receiving elderly donor microbiota showed a ~30% increase in serum I-FABP, indicating that ageing microflora promotes increased intestinal permeability [78]. Studies also indicate that the physical barrier function of the intestinal epithelium may be impacted by ageing. There is evidence that intestinal permeability may increase with age. In older individuals, ileal tissue shows elevated IL-6 levels compared to younger cohorts. Increased claudin-2 also contributes to higher intestinal permeability [79]. Importantly, in the ageing intestinal epithelial stem cells (IESCs), while the expression and distribution of flagellin-specific TLR5 remain unchanged, the intracellular signaling pathways activated upon binding with TLR5 demonstrate age-related alterations. Ageing may influence the loss of intestinal homeostasis through increased activity of c-Jun N-terminal kinase (JNK) in IESCs [80].

Recent experiments show that supplementing beneficial microbes alleviates inflammation, delays ageing, and improves intestinal and liver functions [81]. Early in 2016, van Beek et al. demonstrated that the thickness of the colonic mucus layer, which decreased through the process of ageing, could be restored in ageing by supplementation with *L. plantarum*, a specific lactobacillus strain [82]. Mucolytic bacteria such as *Akkermansia* metabolizes and stimulates the production of mucins, which serve as a protective layer in the gastrointestinal tract, preventing direct microbial interaction with epithelial cells [83]. Certain bacterial strains have been shown to mitigate age-related mucin loss and provide health benefits, such as extending lifespan in progeroid mice and reducing liver dysfunction and inflammatory blood markers [84]. While progeroid mouse models recapitulate certain features of accelerated ageing, they do not fully reflect physiological ageing in humans, limiting direct translational conclusions. For example, supplementing with *Akkermansia* muciniphila reduces inflammation and immune-related processes in older adults, promoting healthy ageing [85]. Studies have shown that FMT from healthy elderly individuals can alter the composition of the gut microbiota, thereby potentially slowing the ageing process [84,86]. While ageing fruit flies show increased gut permeability and reduced lifespan due to changes in their microbiome, data from other animal models, like middle-aged killifish, demonstrate that lifespan can be extended by supplementing with a younger microbiome, highlighting the potential benefits of gut microbiota modulation as a therapeutic strategy for ageing hosts [75]. However, these findings derive from non-mammalian models with distinct gut architecture and immune systems from humans; thus, direct extrapolation to human ageing or ALD requires caution.

### 4.2. Immune Modulation and Inflammation in the Microbiome–Ageing Axis

Changes in microbial composition induce subclinical gut inflammation in older adults, increasing the risk of chronic diseases [87]. Over time, this inflammatory state damages the immune system, hindering its ability to properly clear mutated and ageing cells. Chronic low-grade inflammation is a hallmark of ageing and age-related diseases, with increased inflammatory mediators often resulting from a decline in immune efficiency, known as immunosenescence [88]. Thus, the changes in the gut microbiome are likely regulated by alterations in the immune response associated with ageing, particularly the impact of inflammatory mediators. There is also evidence to suggest that the gut microbiota may play a crucial role in age-related inflammatory responses [89].

Ageing is accompanied by changes in diet, lifestyle and physical activity, as well as a decline in immune function. Compared to healthy younger individuals, the elderly gut displays a distinct loss of diversity-associated taxa [90]. Studies have shown that changes in the composition of the gut microbiome in the elderly are associated with increased levels of inflammatory markers [90,91]. Small intestine biopsies from individuals aged 67–77 show significantly lower IL-8 production by intestinal epithelial cells in response to flagellin from commensal and pathogenic microbes, compared to younger individuals [79]. This suggests that chronic activation of the innate and adaptive immune systems might contribute to changes in the bacterial composition of the gut.

The species that influenced the clustering of age groups included *Muribaculaceae*, *Lachnospiraceae*, *Lactobacillus*, *Clostridium*, and *Prevotella*. In the aged group, *Prevotella* sp., *Lactobacillus intestinalis*, and *Faecalibaculum rodentium* were significantly more abundant, while *Enterorhabdus caecimuris*, *Turicimonas muris*, and *Muribaculaceae bacterium DSM 103720* were enriched in the young group [78]. Interestingly, in healthy individuals aged 70–82 years and an elderly cohort with diabetes or other age-related conditions, no significant gut microbiome changes were detected, except for an increase in the proportion of the genus *Akkermansia* [92]. These findings suggest that the changes in the microbiome may be linked more to the process of ageing itself rather than to age-related illnesses, with specific bacterial taxa potentially contributing to these disorders. In contrast, studying the gut microbiota of centenarians reveals unique patterns. Although centenarians from different regions show variations in microbial composition, influenced by geography, diet, and genetics, there are commonalities distinguishing them from those experiencing unhealthy ageing [93,94,95]. A general increase in microbial species richness is observed. Remarkably, across multiple long-lived groups, there is a decrease in Proteobacteria and an increase in Bacteroidetes. Additionally, the abundance of families like *Lachnospiraceae*, *Ruminococcaceae*, and *Akkermansia* is notably higher in these populations. These findings suggest the presence of universal features in a ‘longevity-associated’ microbial community, offering novel insights into the changes in gut microbiota associated with healthy ageing.

However, although dysbiosis is known to provoke inflammatory responses, changes in the microbiome due to other factors such as diet may also exacerbate inflammation and immune alterations. Thus, it is difficult to interpret whether intestinal inflammation results from microbiota shifts or host-reactive changes, suggesting that immunosenescence and ecological dysregulation may be interdependent.

### 4.3. Microbial Metabolites in the Microbiome–Ageing Axis

Microbial metabolites serve as a crucial functional link connecting the gut microbiota and ageing. Research shows that with ageing, the gut microbiome’s capacity to produce short-chain fatty acids decreases, marked by a loss of associated genes and glycolytic potential, while proteolytic functions become more prominent in older individuals [96]. Notably, these microbial changes are also associated with the plasma levels of inflammatory cytokines such as IL-6 and IL-8. Moreover, research has indicated that with increasing age, there is a reduction in the genes associated with the production of SCFAs, leading to an overall decrease in saccharolytic capacity [96]. Several key microbial metabolites are closely linked to the ageing process, particularly the metabolism of aromatic amino acids such as tryptophan and phenylalanine.

Beyond the direct impacts, ageing also influences the gut microbiota indirectly by affecting the chemical composition of the intestinal mucosa, particularly its glycosylation patterns. Microbes bind to mucin O-glycans via mucin-binding proteins (MUBs), multi-modular cell surface adhesins in lactobacilli, which play a role in bacterial-mucus interactions and colonization. This glycan-mediated interaction between the mucosal layer and gut bacteria is crucial for selecting and maintaining local microbial communities [80]. In germ-free mice receiving microbiota transplants from aged donor mice, researchers observed a decline in *Akkermansia muciniphila*, a mucin-degrading bacterium [97]. This suggests that ageing may thin the mucus layer, increasing intestinal permeability. These alterations could reshape the gut environment, exerting significant effects on the microbial communities and inflammation within the gut. However, research in this area remains in its infancy.

As O’Toole and Jeffery noted, physiological changes in the intestines of older individuals, including diet and physical activity alterations, can impact gut microbiota composition [98]. Ageing is often accompanied by a reduction in the quantity and variety of fiber-rich foods, increasing the risk of malnutrition. Paradoxically, malnutrition may be associated with increased microbial diversity, particularly with an enriched Clostridium sub-group [98]. In recent years, numerous studies have indicated that modulating the gut microbiota through the supplementation of probiotics, prebiotics, and FMT promote the production of beneficial metabolites, such as SCFAs [99]. This optimization of the gut microenvironment enhances microbial diversity, mitigates inflammation, and holds promise for potentially delaying the ageing process.

### 4.4. The Interaction Between Ageing and Intestinal Flora Microbiota Regulates ALD

**Figure 1:**
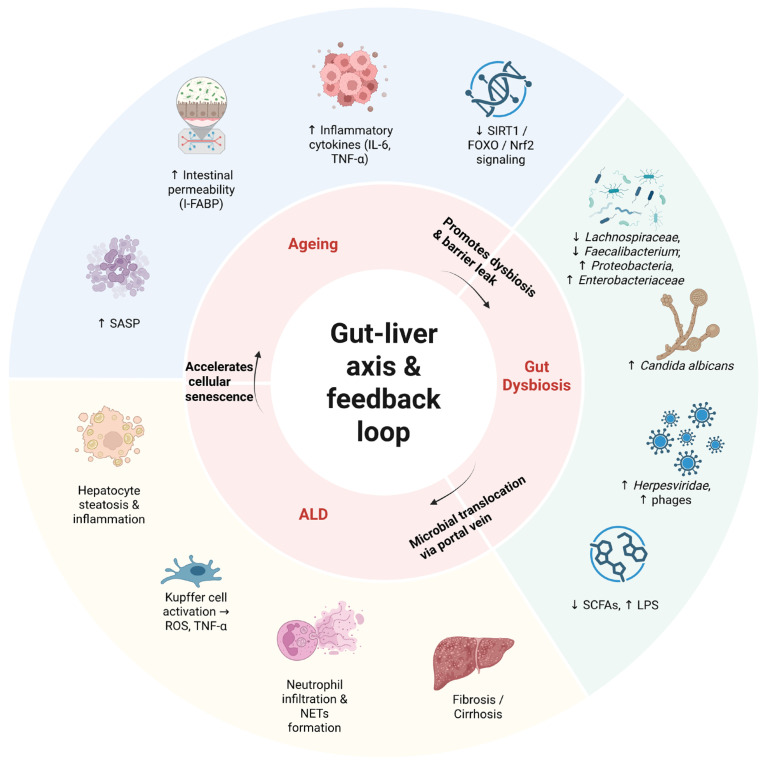
Circular mechanistic model of the ageing–gut microbiota–ALD axis. Ageing downregulates protective SIRT1/FOXO/Nrf2 pathways, increases intestinal permeability, and promotes systemic inflammation, which drives gut dysbiosis characterized by reduced SCFAs, elevated LPS, and overgrowth of pathogenic bacteria, fungi, and viruses. Dysbiosis and barrier disruption allow microbial products to translocate to the liver via the portal vein, exacerbating ALD through hepatocyte injury, Kupffer cell activation, and neutrophil extracellular trap (NET) formation. ALD-induced cellular senescence further accelerates ageing, closing the positive feedback loop. This model highlights that interrupting any node may halt disease progression. Abbreviations: ALD, alcohol-related liver disease; FOXO, forkhead box class O; I-FABP, intestinal fatty acid-binding protein; LPS, lipopolysaccharide; NET, neutrophil extracellular trap; Nrf2, nuclear factor erythroid-derived 2-like 2; SASP, senescence-associated secretory phenotype; SCFA, short-chain fatty acid; SIRT1, sirtuin 1.

It has been recognized that gut microbiota imbalance occurs with age, and its relationship with the development of ageing-related diseases has received increasing attention. In the context of ALD, the interplay between ageing and gut microbiota can be understood through a cascade of interconnected events. Figure 1 presents a conceptual model illustrating this self-reinforcing loop, in which ageing, gut dysbiosis, and ALD progressively amplify one another.

Ageing alters gut microbiota composition and function, which directly contributes to ALD progression. As summarized in Table 2, ageing is characterized by reduced microbial diversity, decreased SCFA-producing taxa, and increased potentially pathogenic bacteria. For instance, with ageing, an increase in harmful bacteria in the gut leads to elevated levels of endotoxins, which in turn activates inflammatory signaling pathways in the liver, exacerbating inflammation and liver fibrosis [77]. Age-related dysbiosis reduces anti-inflammatory metabolites like SCFAs, weakening protection against alcohol-induced liver injury [99]. Simultaneously, the increase in specific pathogenic or harmful bacteria during ageing, such as members of the *Enterobacteriaceae* family, may exacerbate intestinal permeability, promoting inflammation and liver damage [100]. A reduction in *Akkermansia muciniphila*, linked to intestinal mucosa production, is observed in both the elderly and AUD patients, hinting at a connection between the two [14,37,97]. This parallel decline is noteworthy because it suggests that chronic alcohol consumption may drive gut microbiota changes that phenocopy those seen in physiological ageing. These ageing-associated microbial changes compromise the intestinal barrier. Ageing is also accompanied by changes in gut microbiota metabolites, particularly a reduction in SCFAs. These SCFAs play a crucial role in maintaining the structural integrity of the intestinal wall and in regulating immune responses. Consequently, endotoxins and other microbial products translocate across the damaged gut epithelium into the portal circulation, reaching the liver. Once in the liver, these endotoxins activate inflammatory cascades. Ageing and ALD are both accompanied by elevated circulating pro-inflammatory cytokines, including IL-6, IL-1, TNF-α, and C-reactive protein, which are associated with increased morbidity and mortality in liver diseases of the elderly [101,102,103].

Similarly, the gut microbiota influence the expression of ageing genes through their metabolites, thereby promoting the development of ALD. Inhibition of SIRT1 is linked to ageing and ALD, while its upregulation promotes healthy ageing, improving genomic stability and metabolic efficiency [108]. Recent research shows that indole derivatives from gut microbiota alleviate neurodegenerative changes in ageing by activating the GPR30/AMPK/SIRT1 pathway, which also mitigates hepatitis [107]. Additionally, 5-methoxyindoleacetic acid produced by *Lactobacillus rhamnosus GG* has been found to activate Nrf2 in the liver of fruit flies and mice, effectively protecting against oxidative liver damage caused by acute alcohol exposure [109]. Other tryptophan derivatives from gut microbiota, such as indole acetaldehyde and indole-3-acetic acid, have been shown to activate the AhR/Nrf2 pathway, thereby alleviating oxidative stress and endothelial cell damage in the liver [110]. Furthermore, microbial metabolites enhance intestinal barrier integrity and slow ALD progression through the Nrf2 pathway [111]. Transplanting fecal microbiota from young mice to elderly mice reduces inflammatory signals, upregulates the FOXO pathway, and improves gut microbiota composition and metabolome. This significantly lowers the expression of common ageing markers like Vwf, Ehd3, Aspa, Cysltr2, Nt5c3, and Itga6 [112]. Targeting specific strains and ageing pathways could potentially reverse ALD development as research advances.

Consequently, there is a bidirectional interaction between the two. Ageing promotes dysbiosis of the gut microbiota and impaired barrier function, while metabolites produced by the microbiota influence the activity of ageing-related signaling pathways (such as SIRT1, FOXO, and Nrf2), which determine the liver’s sensitivity to alcohol. These interrelated processes synergistically accelerate the development of ALD. Metabolic pathways like the urea cycle, choline metabolism, and bile acid biosynthesis are sensitive to gut microbiome changes, but how these regulate ageing pathways remains unclear, highlighting areas for future research [113,114,115,116]. More studies on the interactions between gut microbiota and ageing in ALD are needed. This is crucial for developing effective treatment strategies. Particularly, those aimed at modulating the gut microbiota and improving intestinal health to mitigate disease progression, thereby providing new targets for clinical intervention.

### 4.5. Causal Hierarchy of the Microbiota–Ageing–ALD Axis

To better distinguish potential driving factors from amplifying factors in the microbiota–ageing–alcoholic liver disease (ALD) axis, we propose an exploratory hierarchical framework organized as “Initial Perturbations → Amplification Loop → End Effects”. It is important to emphasise that this framework serves primarily as a tool for integrating fragmented evidence and generating testable hypotheses, rather than as a definitive description of established causal cascades.

Initial Events (Triggers): Alcohol metabolism generates acetaldehyde and reactive oxygen species (ROS) via CYP2E1, directly causing DNA damage and telomere shortening, and activating the p53/p21 and p16-INK4a-Rb aging pathways in liver cells [54,55]. Oxidative stress damages proteins, nucleic acids, and lipids in the liver, disrupting redox homeostasis and inducing inflammation that aggravates ALD progression [117]. In the intestinal tract, chronic alcohol consumption disrupts epithelial tight junctions, increases mucosal permeability, and promotes bacterial overgrowth and translocation, which initiate gut dysbiosis [8,9]. Concurrently, physiological aging drives immunosenescence through gut bacteria-induced B cell senescence in intestinal germinal centers, resulting in decreased IgA production and diversity, which in turn leads to gut microbiota imbalance [17,118]. However, the chronological distinction among these “initial” events remains ambiguous—ageing and alcohol exposure often occur in parallel or as superimposed hits, and their relative contributions need to be disentangled using longitudinal animal models and birth-cohort studies in humans. Evidence sources: human cohort associations and animal model interventions.

Amplification Cycle (Self-Reinforcing Loop): Gut dysbiosis in ALD is characterized by increased Proteobacteria, decreased Bacteroidetes, reduced SCFA production, and elevated endotoxin levels [119]. Microbial metabolites such as LPS suppress protective aging-related SIRT1/FOXO/Nrf2 pathways, weakening hepatic antioxidant defenses and making hepatocytes more vulnerable to alcohol-induced damage [16,56,57,58]. Conversely, reduced SCFAs impair immune regulation and intestinal barrier integrity, further exacerbating dysbiosis [99]. Moreover, senescence-associated secretory phenotype (SASP) factors (IL-8, IL-17) released from senescent cells can activate Kupffer cells, recruit neutrophils, and promote neutrophil extracellular trap (NET) formation, which releases abundant ROS and proteases that inflict lipid peroxidation upon hepatocytes, inducing secondary senescence in previously non-senescent cells in vitro and in animal models [120]. These interconnected events form a potential positive feedback loop. Importantly, the organism also possesses compensatory stress responses (e.g., transient Nrf2 upregulation and adaptive autophagy activation, i.e., hormesis); under low-dose or early-stage insults, these protective mechanisms may partially offset the damage, and the amplification loop likely dominates only under sustained or high-intensity stimulation. Grape-derived nanovesicles have been shown in preclinical studies to exert hepatoprotective effects by increasing Nrf2 nuclear translocation and upregulating Foxo3a/Sirt1 to induce autophagy, highlighting the therapeutic potential of targeting these pathways [121]. Evidence sources: animal models and cell lines; supporting correlational data from human cohorts.

End Effects (Clinical and Pathological Outcomes): Sustained suppression of SIRT1/FOXO/Nrf2, combined with chronic inflammation and cellular senescence, accelerates the progression from steatosis to fibrosis, cirrhosis, and liver failure [19]. Transcriptomic analysis has revealed increased expression of senescence-associated genes and enrichment of SASP gene signatures in patients with cirrhosis and alcoholic hepatitis, with plasma GDF15 levels correlating with disease severity and 90-day mortality [66]. Targeted clearance of senescent cells has been shown to alleviate ALD by restoring cellular function and immune balance [122]. Microbiota–ageing crosstalk exacerbates ALD severity and increases mortality risk in elderly patients. Evidence: human cohorts, animal models, and in vitro experiments.

Summary of Bidirectional Causality: The relationship between gut microbiota and aging-related genes in ALD is bidirectional and mutually reinforcing. Aging promotes gut dysbiosis through immunosenescence and barrier failure; dysbiosis-derived metabolites suppress SIRT1/FOXO/Nrf2 pathways, accelerating hepatic senescence; senescent hepatocytes release pro-inflammatory factors that further disrupt gut barrier function, closing the loop. Mendelian randomization analysis supports causal relationships among gut microbiota, inflammatory cytokines, and ALD, with inflammatory cytokines potentially acting as mediating factors in the pathway from gut microbiota to ALD [123]. however, it should be noted that this approach captures the cumulative effect of long-term genetic predisposition and cannot resolve the rapid dynamic feedback loops described in our framework. Thus, the bidirectional loop remains largely a hypothetical construct based on correlational and preclinical intervention data. To rigorously test this closed-loop model, future studies should employ combinatorial interventions that simultaneously target microbiota reconstruction and senescent cell clearance, rather than single-pathway blockade experiments. Strength of evidence: most mechanistic insights derive from animal models and in vitro experiments, with supporting correlational data from human cohorts. Definitive causal validation in humans is extremely limited, and this hierarchical framework should be viewed as a tool for guiding experimental design and hypothesis refinement, rather than as a final conclusion on disease mechanisms.

## 5. Conclusions

The pathology of ALD is complex, and growing research suggests a mutual influence between gut microbiota and ageing that may accelerate ALD progression. Studies have shown that the gut microbiota indirectly influences the progression of ALD by regulating ageing-related genes such as SIRT1 and FOXO. Changes in the Firmicutes and Bacteroidetes phyla, along with shifts in metabolites like SCFAs and LPS caused by ageing, profoundly affect the course of ALD. For instance, upregulation of the FOXO pathway has been associated with improve the composition and metabolome of the gut microbiota, which may contribute to alleviating ALD severity. Specifically, FOXO activation enhances hepatic and systemic antioxidative defenses by upregulating superoxide dismutase (SOD) and catalase (CAT), thereby reducing oxidative stress and systemic inflammation that otherwise drives dysbiosis. By extension, this could in theory mitigate the inflammatory drive that promotes dysbiosis, although direct evidence for this causal sequence in ALD models remains limited. Second, by suppressing pro-inflammatory cytokine production and, in some contexts, neutrophil extracellular trap (NET) formation, which may help control chronic inflammation and limit pathobiont expansion, yet these observations derive largely from non-ALD settings (e.g., adipocytes and neurodegenerative models) and await verification in alcohol-related liver injury. Third, FOXO may influence intestinal barrier integrity, potentially through modulation of tight junction proteins and mucin production, but this putative mechanism has not been directly demonstrated in ALD. And fourth, through its interplay with Nrf2 and AMPK pathways, FOXO could theoretically influence host metabolic outputs (including bile acid profiles and short-chain fatty acid utilization) that selectively shape microbial composition, though this remains speculative. Collectively, while these interconnected pathways offer a plausible framework, direct causal evidence in ALD is currently lacking, and caution is warranted when extrapolating these findings to clinical translation. Conversely, downregulation of ageing pathways like FOXO and Nrf2 alters gut microbiota composition and diversity, worsening the disease. The interplay between gut microbiota and ageing pathways not only accelerates ALD progression but also offers new insights for disease treatment. Future research should focus on the mechanisms of interaction between gut microbiota and ageing pathways, targeting them as potential strategies for ALD intervention. This will not only enhance our understanding of the pathogenesis of ALD but also provide potential targets for clinical treatment.

While animal models offer valuable insights, their relevance to human conditions remains uncertain. Given the complexity of gut microbiota and its regulation by environmental factors, the effects of microbial communities on both ageing and ALD are still unclear. Future studies should emphasize understanding the roles of bacterial strains, microbial genes, and metabolites in healthy ageing. This could facilitate the development of personalized treatments and new therapeutic strategies. Moreover, probiotics, particularly genetically engineered strains, show great potential as treatment strategies due to their ability to precisely deliver therapeutic molecules. However, the efficacy of these approaches requires further validation through extensive clinical trials, especially with promising microbes such as *Lactobacillus* and *Akkermansia muciniphila*. Additionally, interventions targeting the intestinal barrier are critical for improving ALD outcomes. Regulating gut microbiota to maintain intestinal barrier integrity slows ageing and enhances liver function. To advance the transition from mechanistic understanding to therapeutic translation, we propose the following specific research priorities based on the interaction between the microbiome and ageing: (1) Validate age-related microbial signatures as predictors of ALD progression. Longitudinal cohort studies are needed to determine whether baseline gut microbiota composition in middle-aged and older adults can predict the severity of alcoholic liver damage independently of alcohol consumption levels. Specifically, prospective cohorts with age stratification (e.g., <50, 50–65, and >65 years) should be designed to clarify whether microbial biomarkers, such as reduced Faecalibacterium, elevated Enterobacteriaceae, or decreased SCFA producers, can predict ALD progression in elderly populations. (2) Most current studies on ALD use young animals; future experiments should specifically evaluate probiotics, prebiotics, and FMT in aged or geriatric mouse models subjected to chronic, high-dose ethanol exposure. Priority should be given to testing FMT from healthy young donors and specific probiotic strains (e.g., *Akkermansia muciniphila*, *Lactobacillus rhamnosus* GG) in aged ALD models, with outcomes including intestinal permeability, hepatic inflammation, and fibrosis. (3) Although correlations exist between short-chain fatty acids, tryptophan derivatives, and SIRT1/FOXO/Nrf2 activity, causal evidence still requires germ-free animal models and pathway-specific inhibitors or activators. (4) Probiotic or postbiotic candidates that demonstrate efficacy in aged animal models should be evaluated in early-phase clinical trials, with outcome measures clearly stratified by age group. (5) Beyond single interventions, combined strategies targeting both the gut microbiota and host ageing pathways warrant investigation. For instance, the synergistic effects of SIRT1 agonists (e.g., nicotinamide riboside) administered together with probiotics or FMT should be explored in aged ALD models, as concurrent reinforcement of microbial homeostasis and host defence mechanisms may yield greater therapeutic benefit than either approach alone. Implementing these strategies will provide a clearer understanding of the role of the gut microbiota and ageing in the progression of ALD, offering new insights for delaying disease onset and improving the quality of life for high-risk older adults.

## Figures and Tables

**Table 1:** Summary of Gut Microbiota Alterations in Alcohol-Related Liver Disease.

References	Study Subjects (Population/Model)	Methods	Key Findings (Microbial Changes)
Bacteria			
Yang et al. (2025) [27]	AH patients & mouse models	Metagenomic sequencing, germ-free mouse colonization, bacterial genetic manipulation	Increase: *Escherichia coli* strains carrying the KpsM virulence factor; abundance correlates with patient mortality.
Zhang et al. (2025) [28]	ALD patients & ethanol-fed mice	16S rDNA sequencing, metabolomics, proteomics	Decrease: *Lachnospiraceae*; abundance negatively correlates with liver injury markers (ALT, AST). Supplementation alleviated liver disease.
Yin et al. (2025) [29]	Mouse models of ALD	Metagenomic data analysis, culturomics	Increase: The abundance of the butyrate-producing bacterium *Faecalibaculum rodentium* and butyrate content in the intestinal lumen. Decrease: Ethanol-induced overgrowth of conditional intestinal pathogens.
Zhang et al. (2023) [30]	WT and Ppara-null mice with ALD	16S rRNA sequencing, Liver and Urine Metabolomics	Increase: *Clostridiumsensustricto1* and *Romboutsia*; Decrease: *Helicobacter*, *Monoglobus*, and *Bacteroides*.
**Fungi**			
Chu et al. (2020) [31]	AH vs. Controls	quantitative PCR	Increase: *Candida albicans*
Lang et al. (2020) [32]	AH, AUD, and Controls	Fungal ITS sequencing of fecal samples; Serum ASCA measurement (ELISA)	Increase: *Candida* Decrease: *Penicillium*
**Virus**			
Jiang et al. (2020) [33]	AH, AUD, and Controls	Metagenomic sequencing of VLPs from fecal samples	Phages: Increase: Escherichia-, Enterobacteria-, Enterococcus-, Lactobacillus-, and Staphylococcus phages. Mammalian Viruses: Increase: Herpesviridae, Parvoviridae.

**Table note:** Studies included in this table were selected from peer-reviewed articles utilizing high-throughput sequencing (16S rRNA, metagenomics, ITS, or viral metagenomics) in human subjects or animal models of alcohol-related liver disease. **Abbreviation**: AH, alcohol-related hepatitis; ALD, alcohol-related liver disease; ALT, alanine transaminase; AST, aspartate transaminase; WT, wild-type; Ppara, peroxisome proliferator-activated receptor alpha; ITS, internal transcribed spacer; AUD, alcohol use disorder; ASCA, anti-saccharomyces cerevisiae antibody; ELISA, enzyme-linked immunosorbent assay; VLPs, virus-like particles.

**Table 2:** Summary of Gut Microbiota Alterations in Ageing and Longevity.

References	Study Subjects (Population/Model)	Methods	Key Findings (Microbial Changes)
Bacteria			
Bradley & Haran (2024) [18]	Humans across lifespan (1–100+ years)	Narrative review	Decrease: diversity after age 65, especially >80 years. Healthy ageing associated with diverse microbiome enriched in anti-inflammatory metabolite producers (e.g., SCFA producers).
Xiao et al. (2025) [104]	Review of ageing-associated microbiota	Comprehensive review	Decrease: *Lachnospiraceae*, *Ruminococcaceae*, *Faecalibacterium*, *Roseburia*. Increase: *Akkermansia muciniphila*, *Christensenellaceae* in centenarians; core longevity-associated taxa include *Eisenbergiella tayi*, *Methanobrevibacter smithii*, *Desulfovibrio fairfieldensis*.
Chen et al. (2024) [105]	Long-lived populations vs. elderly & young adults	Fecal metagenomics + Mendelian randomization	Increase in long-lived individuals: *Eisenbergiella tayi*, *Methanobrevibacter smithii*, *Hungatella hathewayi*, *Desulfovibrio fairfieldensis*; *E. tayi* involved in protein N-glycosylation.
Parker et al. (2022) [78]	Young vs. aged mice	FMT, 16S rRNA sequencing	Serum I-FABP increased ~30% in young mice receiving elderly donor microbiota; aged gut promoted intestinal permeability. Aged group enriched: *Prevotella* sp., *Lactobacillus intestinalis*, *Faecalibaculum rodentium*. Young group enriched: *Enterorhabdus caecimuris*, *Turicimonas muris*, *Muribaculaceae*.
**Fungi/Archaea**			
Pang et al. (2023) [106]	Centenarians (20–117 years, *n* = 1575)	Metagenomic sequencing	Gut mycobiome develops with ageing; long-lived individuals possess unique fungal signatures with “youth-associated” microbial patterns.
**Metabolites**			
Yin et al. (2023) [107]	Ageing models	Metabolite analysis	Gut microbiota-derived indole derivatives activate GPR30/AMPK/SIRT1 pathway, alleviating neurodegeneration in ageing.
Hou et al. (2023) [99]	Ageing models	Dietary intervention	Dietary genistein increases microbiota-derived SCFA levels, modulates gut homeostasis, extends healthspan and lifespan.

**Table note:** Studies selected from peer-reviewed articles (2022–2025) on gut microbiota alterations associated with ageing, frailty, or longevity. Abbreviations: FMT, fecal microbiota transplantation; SCFA, short-chain fatty acid.

## Data Availability

No new data were created or analyzed in this study. Data sharing is not applicable to this article.

## Abbreviations

The following abbreviations are used in this manuscript:

ADH	Alcohol Dehydrogenase
ALD	Alcohol-Related Liver Disease
ALDH	Aldehyde Dehydrogenase
ALT	Alanine Transaminase
ASCA	Anti-Saccharomyces cerevisiae Antibody
AST	Aspartate Transaminase
AUD	Alcohol Use Disorder
CYP2E1	Cytochrome P450 2E1
ELISA	Enzyme-Linked Immunosorbent Assay
FOXO	Forkhead Box, Class O
FMT	Fecal Microbiota Transplantation
I-FABP	Intestinal Fatty Acid-Binding Protein
IESCs	Intestinal Epithelial Stem Cells
IL	Interleukin
ITS	Internal Transcribed Spacer
JNK	c-Jun N-Terminal Kinase
LPS	Lipopolysaccharide
MUBs	Mucin-Binding Proteins
NAD	Nicotinamide Adenine Dinucleotide
NET	Neutrophil Extracellular Trap
NR	Nicotinamide Riboside
Nrf2	Nuclear Factor Erythroid-Derived 2-Like 2
Ppara	Peroxisome Proliferator-Activated Receptor Alpha
ROS	Reactive Oxygen Species
SASP	Senescence-Associated Secretory Phenotype
SCFAs	Short-Chain Fatty Acids
SIRT1	Sirtuin 1
SOD	Superoxide Dismutase
TNF-α	Tumor Necrosis Factor Alpha
VLPs	Virus-Like Particles
WT	Wild-Type

## References

[1] Åberg F., Jiang Z.G., Cortez-Pinto H., Männistö V. (2024). Alcohol-Associated Liver Disease-Global Epidemiology. Hepatology.

[2] Alvarado-Tapias E., Pose E., Gratacós-Ginès J., Clemente-Sánchez A., López-Pelayo H., Bataller R. (2025). Alcohol-Associated Liver Disease: Natural History, Management and Novel Targeted Therapies. Clin. Mol. Hepatol..

[3] Nagarjuna D., Karthikeyan E. (2025). Alcohol-Associated Liver Disease: A Review. Gastroenterol. Endosc..

[4] Devarbhavi H., Asrani S.K., Arab J.P., Nartey Y.A., Pose E., Kamath P.S. (2023). Global Burden of Liver Disease: 2023 Update. J. Hepatol..

[5] Hagström H., Thiele M., Roelstraete B., Söderling J., Ludvigsson J.F. (2021). Mortality in Biopsy-Proven Alcohol-Related Liver Disease: A Population-Based Nationwide Cohort Study of 3453 Patients. Gut.

[6] Liu T., Zhang F., Feng Y., Han P., Gao Y. (2025). Alcohol-Metabolizing Enzymes, Liver Diseases and Cancer. Semin. Liver Dis..

[7] Maccioni R., Tambaro S., Doro L., Bassareo V., Peana A.T., Acquas E. (2026). Timeless and Stainless Alcohol: Concentric Waves from Its Oxidative Metabolism and Related Oxidative Stress. Antioxidants.

[8] Kuo C.-H., Wu L.-L., Chen H.-P., Yu J., Wu C.-Y. (2024). Direct Effects of Alcohol on Gut-Epithelial Barrier: Unraveling the Disruption of Physical and Chemical Barrier of the Gut-Epithelial Barrier That Compromises the Host-Microbiota Interface upon Alcohol Exposure. J. Gastroenterol. Hepatol..

[9] Sosnowski K., Przybyłkowski A. (2024). Ethanol-Induced Changes to the Gut Microbiome Compromise the Intestinal Homeostasis: A Review. Gut Microbes.

[10] Jophlin L.L., Singal A.K., Bataller R., Wong R.J., Sauer B.G., Terrault N.A., Shah V.H. (2024). ACG Clinical Guideline: Alcohol-Associated Liver Disease. Am. J. Gastroenterol..

[11] Mackowiak B., Fu Y., Maccioni L., Gao B. (2024). Alcohol-Associated Liver Disease. J. Clin. Investig..

[12] Bajaj J.S., Heuman D.M., Hylemon P.B., Sanyal A.J., White M.B., Monteith P., Noble N.A., Unser A.B., Daita K., Fisher A.R. (2014). Altered Profile of Human Gut Microbiome Is Associated with Cirrhosis and Its Complications. J. Hepatol..

[13] Fan Y., Pedersen O. (2021). Gut Microbiota in Human Metabolic Health and Disease. Nat. Rev. Microbiol..

[14] Addolorato G., Ponziani F.R., Dionisi T., Mosoni C., Vassallo G.A., Sestito L., Petito V., Picca A., Marzetti E., Tarli C. (2020). Gut Microbiota Compositional and Functional Fingerprint in Patients with Alcohol Use Disorder and Alcohol-Associated Liver Disease. Liver Int..

[15] Smirnova E., Puri P., Muthiah M.D., Daitya K., Brown R., Chalasani N., Liangpunsakul S., Shah V.H., Gelow K., Siddiqui M.S. (2020). Fecal Microbiome Distinguishes Alcohol Consumption from Alcoholic Hepatitis but Does Not Discriminate Disease Severity. Hepatology.

[16] Ramirez T., Li Y.-M., Yin S., Xu M.-J., Feng D., Zhou Z., Zang M., Mukhopadhyay P., Varga Z.V., Pacher P. (2017). Aging Aggravates Alcoholic Liver Injury and Fibrosis in Mice by Downregulating Sirtuin 1 Expression. J. Hepatol..

[17] Kawamoto S., Uemura K., Hori N., Takayasu L., Konishi Y., Katoh K., Matsumoto T., Suzuki M., Sakai Y., Matsudaira T. (2023). Bacterial Induction of B Cell Senescence Promotes Age-Related Changes in the Gut Microbiota. Nat. Cell Biol..

[18] Bradley E., Haran J. (2024). The Human Gut Microbiome and Aging. Gut Microbes.

[19] Sanfeliu-Redondo D., Gibert-Ramos A., Gracia-Sancho J. (2024). Cell Senescence in Liver Diseases: Pathological Mechanism and Theranostic Opportunity. Nat. Rev. Gastroenterol. Hepatol..

[20] Gao H., Nepovimova E., Heger Z., Valko M., Wu Q., Kuca K., Adam V. (2023). Role of Hypoxia in Cellular Senescence. Pharmacol. Res..

[21] Sarin S.K., Pande A., Schnabl B. (2019). Microbiome as a Therapeutic Target in Alcohol-Related Liver Disease. J. Hepatol..

[22] Ganesan R., Thirumurugan D., Vinayagam S., Kim D.J., Suk K.T., Iyer M., Yadav M.K., HariKrishnaReddy D., Parkash J., Wander A. (2025). A Critical Review of Microbiome-Derived Metabolic Functions and Translational Research in Liver Diseases. Front. Cell. Infect. Microbiol..

[23] Bajaj J.S. (2019). Alcohol, Liver Disease and the Gut Microbiota. Nat. Rev. Gastroenterol. Hepatol..

[24] Hartmann P., Lang S., Schierwagen R., Klein S., Praktiknjo M., Trebicka J., Schnabl B. (2023). Fecal Cytolysin Does Not Predict Disease Severity in Acutely Decompensated Cirrhosis and Acute-on-Chronic Liver Failure. Hepatobiliary Pancreat. Dis. Int..

[25] Vidya Bernhardt G., Shivappa P., Pinto J.R., Ks R., Ramakrishna Pillai J., Kumar Srinivasamurthy S., Paul Samuel V. (2024). Probiotics-Role in Alleviating the Impact of Alcohol Liver Disease and Alcohol Deaddiction: A Systematic Review. Front. Nutr..

[26] Lv J., Lang G., Wang Q., Zhao W., Shi D., Zhou Z., Shen Y., Xia H., Han S., Li L. (2024). *Lactobacillus helveticus* Attenuates Alcoholic Liver Injury via Regulation of Gut Microecology in Mice. Microb. Biotechnol..

[27] Yang Y.Q., Duan Y., Lang S., Fondevila M.F., Schöler D., Harberts A., Cabré N., Chen S.N., Shao Y., Vervier K. (2025). Targeted Inhibition of Pathobiont Virulence Factor Mitigates Alcohol-Associated Liver Disease. Cell Host Microbe.

[28] Zhang H., Hu Q., Zhang Y., Yang L., Tian S., Zhang X., Shen H., Shu H., Xie L., Wu D. (2025). *Lachnospiraceae bacterium* Alleviates Alcohol-Associated Liver Disease by Enhancing N-Acetyl-Glutamic Acid Levels and Inhibiting Ferroptosis through the KEAP1-NRF2 Pathway. Gut Microbes.

[29] Yin R., Wang T., Sun J., Dai H., Zhang Y., Liu N., Liu H. (2025). Postbiotics from *Lactobacillus johnsonii* Activates Gut Innate Immunity to Mitigate Alcohol-Associated Liver Disease. Adv. Sci..

[30] Zhang T., Bao L., Zhao Q., Wu Z.E., Dai M., Rao Q., Li F. (2023). Metabolomics Reveals Gut Microbiota Contribute to PPARα Deficiency-Induced Alcoholic Liver Injury. J. Proteome Res..

[31] Chu H., Duan Y., Lang S., Jiang L., Wang Y., Llorente C., Liu J., Mogavero S., Bosques-Padilla F., Abraldes J.G. (2020). The *Candida albicans* Exotoxin Candidalysin Promotes Alcohol-Associated Liver Disease. J. Hepatol..

[32] Lang S., Duan Y., Liu J., Torralba M.G., Kuelbs C., Ventura-Cots M., Abraldes J.G., Bosques-Padilla F., Verna E.C., Brown R.S. (2020). Intestinal Fungal Dysbiosis and Systemic Immune Response to Fungi in Patients With Alcoholic Hepatitis. Hepatology.

[33] Jiang L., Lang S., Duan Y., Zhang X., Gao B., Chopyk J., Schwanemann L.K., Ventura-Cots M., Bataller R., Bosques-Padilla F. (2020). Intestinal Virome in Patients With Alcoholic Hepatitis. Hepatology.

[34] Kuo C.-H., El-Omar E., Kao C.-Y., Lin J.-T., Wu C.-Y. (2025). Compositional and Metabolomic Shifts of the Gut Microbiome in Alcohol-Related Liver Disease. J. Gastroenterol. Hepatol..

[35] Ciocan D., Voican C.S., Wrzosek L., Hugot C., Rainteau D., Humbert L., Cassard A.-M., Perlemuter G. (2018). Bile Acid Homeostasis and Intestinal Dysbiosis in Alcoholic Hepatitis. Aliment. Pharmacol. Ther..

[36] Yang F., Zhao Y.-E., Wei J.-D., Lu Y.-F., Zhang Y., Sun Y.-L., Ma M.-Y., Zhang R.-L. (2018). Comparison of Microbial Diversity and Composition in Jejunum and Colon of the Alcohol-Dependent Rats. J. Microbiol. Biotechnol..

[37] Dubinkina V.B., Tyakht A.V., Odintsova V.Y., Yarygin K.S., Kovarsky B.A., Pavlenko A.V., Ischenko D.S., Popenko A.S., Alexeev D.G., Taraskina A.Y. (2017). Links of Gut Microbiota Composition with Alcohol Dependence Syndrome and Alcoholic Liver Disease. Microbiome.

[38] Kirpich I.A., Solovieva N.V., Leikhter S.N., Shidakova N.A., Lebedeva O.V., Sidorov P.I., Bazhukova T.A., Soloviev A.G., Barve S.S., McClain C.J. (2008). Probiotics Restore Bowel Flora and Improve Liver Enzymes in Human Alcohol-Induced Liver Injury: A Pilot Study. Alcohol.

[39] Tuomisto S., Pessi T., Collin P., Vuento R., Aittoniemi J., Karhunen P.J. (2014). Changes in Gut Bacterial Populations and Their Translocation into Liver and Ascites in Alcoholic Liver Cirrhotics. BMC Gastroenterol..

[40] Gao W., Zhu Y., Ye J., Chu H. (2021). Gut Non-Bacterial Microbiota Contributing to Alcohol-Associated Liver Disease. Gut Microbes.

[41] Zeng S., Rosati E., Saggau C., Messner B., Chu H., Duan Y., Hartmann P., Wang Y., Ma S., Huang W.J.M. (2023). *Candida albicans*-Specific Th17 Cell-Mediated Response Contributes to Alcohol-Associated Liver Disease. Cell Host Microbe.

[42] Yang A.-M., Inamine T., Hochrath K., Chen P., Wang L., Llorente C., Bluemel S., Hartmann P., Xu J., Koyama Y. (2017). Intestinal Fungi Contribute to Development of Alcoholic Liver Disease. J. Clin. Investig..

[43] Zeng S., Schnabl B. (2022). Roles for the Mycobiome in Liver Disease. Liver Int..

[44] Chen L., Zhu Y., Hou X., Yang L., Chu H. (2022). The Role of Gut Bacteria and Fungi in Alcohol-Associated Liver Disease. Front. Med..

[45] Duan Y., Llorente C., Lang S., Brandl K., Chu H., Jiang L., White R.C., Clarke T.H., Nguyen K., Torralba M. (2019). Bacteriophage Targeting of Gut Bacterium Attenuates Alcoholic Liver Disease. Nature.

[46] Kim S.S., Eun J.W., Cho H.J., Song D.S., Kim C.W., Kim Y.S., Lee S.W., Kim Y.K., Yang J., Choi J. (2021). Microbiome as a Potential Diagnostic and Predictive Biomarker in Severe Alcoholic Hepatitis. Aliment. Pharmacol. Ther..

[47] Lang S., Fairfied B., Gao B., Duan Y., Zhang X., Fouts D.E., Schnabl B. (2020). Changes in the Fecal Bacterial Microbiota Associated with Disease Severity in Alcoholic Hepatitis Patients. Gut Microbes.

[48] Gao B., Emami A., Nath S., Schnabl B. (2021). Microbial Products and Metabolites Contributing to Alcohol-Related Liver Disease. Mol. Nutr. Food Res..

[49] Zeng S., Hartmann P., Park M., Duan Y., Lang S., Llorente C., Wang Y., Cabré N., Fouts D.E., Bacher P. (2023). *Malassezia restricta* Promotes Alcohol-Induced Liver Injury. Hepatol. Commun..

[50] Viebahn G., Hartmann P., Lang S., Demir M., Zhang X., Fouts D.E., Stärkel P., Schnabl B. (2024). Fungal Signature Differentiates Alcohol-Associated Liver Disease from Nonalcoholic Fatty Liver Disease. Gut Microbes.

[51] Crunkhorn S. (2019). Phages Fight Alcoholic Hepatitis. Nat. Rev. Drug Discov..

[52] Ren R., He Y., Ding D., Cui A., Bao H., Ma J., Hou X., Li Y., Feng D., Li X. (2022). Aging Exaggerates Acute-on-Chronic Alcohol-Induced Liver Injury in Mice and Humans by Inhibiting Neutrophilic Sirtuin 1-C/EBPα-miRNA-223 Axis. Hepatology.

[53] Liang H., Ding X., Liu S., Tong S., Wang X., Zhang Z., Wang W., Zhang X., Yuan Y., Jiang Y. (2026). Aging-Caused the Changes of the Gut Microbiota Drive Intestinal Barrier Dysfunction and Increase Sepsis Susceptibility. Gut Microbes.

[54] Oka Y., Nakazawa Y., Shimada M., Ogi T. (2024). Endogenous Aldehyde-Induced DNA-Protein Crosslinks Are Resolved by Transcription-Coupled Repair. Nat. Cell Biol..

[55] Du K., Umbaugh D.S., Ren N., Diehl A.M. (2026). Cellular Senescence in Liver Diseases: From Molecular Drivers to Therapeutic Targeting. J. Hepatol..

[56] Jiang Y., Wei S., Shen S., Liu Y., Su W., Ding D., Zheng Z., Yu H., Zhang T., Yang Q. (2025). Ethyl Lactate Ameliorates Hepatic Steatosis and Acute-on-Chronic Liver Injury in Alcohol-Associated Liver Disease by Inducing Fibroblast Growth Factor 21. Adv. Sci..

[57] Klotz L.-O., Sánchez-Ramos C., Prieto-Arroyo I., Urbánek P., Steinbrenner H., Monsalve M. (2015). Redox Regulation of FoxO Transcription Factors. Redox Biol..

[58] Sun X., Wang P., Yao L.-P., Wang W., Gao Y.-M., Zhang J., Fu Y.-J. (2018). Paeonol Alleviated Acute Alcohol-Induced Liver Injury via SIRT1/Nrf2/NF-κB Signaling Pathway. Environ. Toxicol. Pharmacol..

[59] Chembazhi U.V., Bangru S., Dutta R., Das D., Peiffer B., Natua S., Toohill K., Leona A., Purwar I., Bhowmik A. (2024). Dysregulated RNA Splicing Induces Regeneration Failure in Alcohol-Associated Liver Disease. bioRxiv.

[60] Petagine L., Zariwala M.G., Patel V.B. (2021). Alcoholic Liver Disease: Current Insights into Cellular Mechanisms. World J. Biol. Chem..

[61] Greenlee-Wacker M.C. (2016). Clearance of Apoptotic Neutrophils and Resolution of Inflammation. Immunol. Rev..

[62] Cho Y., Bukong T.N., Tornai D., Babuta M., Vlachos I.S., Kanata E., Catalano D., Szabo G. (2023). Neutrophil Extracellular Traps Contribute to Liver Damage and Increase Defective Low-Density Neutrophils in Alcohol-Associated Hepatitis. J. Hepatol..

[63] Liu K., Wang F.-S., Xu R. (2021). Neutrophils in Liver Diseases: Pathogenesis and Therapeutic Targets. Cell. Mol. Immunol..

[64] Xu M., Xu H., Ling Y.-W., Liu J.-J., Song P., Fang Z.-Q., Yue Z.-S., Duan J.-L., He F., Wang L. (2026). Neutrophil Extracellular Traps-Triggered Hepatocellular Senescence Exacerbates Lipotoxicity in Non-Alcoholic Steatohepatitis. J. Adv. Res..

[65] Rodrigo-Torres D., Kilpatrick A.M., Ferreira-Gonzalez S., Aird R.E., Atkinson S.R., Gadd V.L., Man T.Y., Tyson L.D., Dhondalay G.K.R., Vergis N. (2025). Longitudinal Paired Liver Biopsies and Transcriptome Profiling in Alcohol-Associated Hepatitis Reveal Dynamic Changes in Cellular Senescence. Gut.

[66] Rubio-Tomás T., Martí-Aguado D., Blaya D., Ariño S., Aguilar-Bravo B., Martínez García de la Torre R.A., Miravet-Marti M., Ferrer-Lorente R., Zanatto L., Xu Z. (2025). GDF15 Is Associated with Hepatocellular Senescence and Correlates with Mortality in Patients with Alcohol-Associated Hepatitis. JHEP Rep. Innov. Hepatol..

[67] Wang S.F., Wan T., Ye M.T., Qiu Y., Pei L., Jiang R., Pang N.Z., Huang Y.L., Liang B.X., Ling W.H. (2018). Nicotinamide Riboside Attenuates Alcohol Induced Liver Injuries via Activation of SirT1/PGC-1α/Mitochondrial Biosynthesis Pathway. Redox Biol..

[68] Qu Q., Chen Y., Wang Y., Wang W., Long S., Yang H.-Y., Wu J., Li M., Tian X., Wei X. (2025). Lithocholic Acid Binds TULP3 to Activate Sirtuins and AMPK to Slow down Ageing. Nature.

[69] Yang L., Liu D., Jiang S., Li H., Chen L., Wu Y., Essien A.E., Opoku M., Naranmandakh S., Liu S. (2024). SIRT1 Signaling Pathways in Sarcopenia: Novel Mechanisms and Potential Therapeutic Targets. Biomed. Pharmacother..

[70] Heo M.J., Kim T.H., You J.S., Blaya D., Sancho-Bru P., Kim S.G. (2019). Alcohol Dysregulates miR-148a in Hepatocytes through FoxO1, Facilitating Pyroptosis via TXNIP Overexpression. Gut.

[71] Sun J., Hong Z.X., Shao S., Li L., Yang B., Hou Y.Y., Wang H.H., Xu Y.Y., Zhang Q., Pi J.B. (2021). Liver-Specific Nrf2 Deficiency Accelerates Ethanol-Induced Lethality and Hepatic Injury in Vivo. Toxicol. Appl. Pharmacol..

[72] Bardag-Gorce F., Oliva J., Lin A., Li J., French B.A., French S.W. (2011). Proteasome Inhibitor up Regulates Liver Antioxidative Enzymes in Rat Model of Alcoholic Liver Disease. Exp. Mol. Pathol..

[73] Xu L., Yu Y.F., Sang R., Li J.X., Ge B.J., Zhang X.M. (2018). Protective Effects of Taraxasterol against Ethanol-induced Liver Injury by Regulating CYP2E1/Nrf2/HO-1 and NF-*κ*B Signaling Pathways in Mice. Oxid. Med. Cell. Longev..

[74] Cox A.J., West N.P., Cripps A.W. (2015). Obesity, Inflammation, and the Gut Microbiota. Lancet Diabetes Endocrinol..

[75] Clark R.I., Salazar A., Yamada R., Fitz-Gibbon S., Morselli M., Alcaraz J., Rana A., Rera M., Pellegrini M., Ja W.W. (2015). Distinct Shifts in Microbiota Composition during *Drosophila* Aging Impair Intestinal Function and Drive Mortality. Cell Rep..

[76] Rashidah N.H., Lim S.M., Neoh C.F., Majeed A.B.A., Tan M.P., Khor H.M., Tan A.H., Rehiman S.H., Ramasamy K. (2022). Differential Gut Microbiota and Intestinal Permeability between Frail and Healthy Older Adults: A Systematic Review. Ageing Res. Rev..

[77] Fatima S., Altwaijry H., Abulmeaty M.M.A., Abudawood M., Siddiqi N.J., Alrashoudi R.H., Alsobaie S. (2023). Combined Supplementation of *Clostridium butyricum* and *Bifidobacterium infantis* Diminishes Chronic Unpredictable Mild Stress-Induced Intestinal Alterations via Activation of Nrf-2 Signaling Pathway in Rats. Int. J. Mol. Sci..

[78] Parker A., Romano S., Ansorge R., Aboelnour A., Le Gall G., Savva G.M., Pontifex M.G., Telatin A., Baker D., Jones E. (2022). Fecal Microbiota Transfer between Young and Aged Mice Reverses Hallmarks of the Aging Gut, Eye, and Brain. Microbiome.

[79] Man A.L., Bertelli E., Rentini S., Regoli M., Briars G., Marini M., Watson A.J.M., Nicoletti C. (2015). Age-Associated Modifications of Intestinal Permeability and Innate Immunity in Human Small Intestine. Clin. Sci..

[80] Branca J.J.V., Gulisano M., Nicoletti C. (2019). Intestinal Epithelial Barrier Functions in Ageing. Ageing Res. Rev..

[81] Li H.J., Qi Y.Y., Jasper H. (2016). Preventing Age-Related Decline of Gut Compartmentalization Limits Microbiota Dysbiosis and Extends Lifespan. Cell Host Microbe.

[82] Van Beek A.A., Sovran B., Hugenholtz F., Meijer B., Hoogerland J.A., Mihailova V., Van Der Ploeg C., Belzer C., Boekschoten M.V., Hoeijmakers J.H.J. (2016). Supplementation with *Lactobacillus plantarum* WCFS1 Prevents Decline of Mucus Barrier in Colon of Accelerated Aging Ercc1−/Δ7 Mice. Front. Immunol..

[83] Dieterich W., Schink M., Zopf Y. (2018). Microbiota in the Gastrointestinal Tract. Med. Sci..

[84] Bárcena C., Valdés-Mas R., Mayoral P., Garabaya C., Durand S., Rodríguez F., Fernández-García M.T., Salazar N., Nogacka A.M., Garatachea N. (2019). Healthspan and Lifespan Extension by Fecal Microbiota Transplantation into Progeroid Mice. Nat. Med..

[85] van der Lugt B., van Beek A.A., Aalvink S., Meijer B., Sovran B., Vermeij W.P., Brandt R.M.C., de Vos W.M., Savelkoul H.F.J., Steegenga W.T. (2019). Akkermansia Muciniphila Ameliorates the Age-Related Decline in Colonic Mucus Thickness and Attenuates Immune Activation in Accelerated Aging Ercc1−/Δ7 Mice. Immun. Ageing.

[86] Philips C.A., Ahamed R., Rajesh S., Abduljaleel J.K.P., Augustine P. (2022). Long-Term Outcomes of Stool Transplant in Alcohol-Associated Hepatitis—Analysis of Clinical Outcomes, Relapse, Gut Microbiota and Comparisons with Standard Care. J. Clin. Exp. Hepatol..

[87] Guigoz Y., Doré J., Schiffrin E.J. (2008). The Inflammatory Status of Old Age Can Be Nurtured from the Intestinal Environment. Curr. Opin. Clin. Nutr. Metab. Care.

[88] Baechle J.J., Chen N., Makhijani P., Winer S., Furman D., Winer D.A. (2023). Chronic Inflammation and the Hallmarks of Aging. Mol. Metab..

[89] Cattaneo A., Cattane N., Galluzzi S., Provasi S., Lopizzo N., Festari C., Ferrari C., Guerra U.P., Paghera B., Muscio C. (2017). Association of Brain Amyloidosis with Pro-Inflammatory Gut Bacterial Taxa and Peripheral Inflammation Markers in Cognitively Impaired Elderly. Neurobiol. Aging.

[90] Claesson M.J., Jeffery I.B., Conde S., Power S.E., O’Connor E.M., Cusack S., Harris H.M.B., Coakley M., Lakshminarayanan B., O’Sullivan O. (2012). Gut Microbiota Composition Correlates with Diet and Health in the Elderly. Nature.

[91] Franceschi C., Garagnani P., Parini P., Giuliani C., Santoro A. (2018). Inflammaging: A New Immune–Metabolic Viewpoint for Age-Related Diseases. Nat. Rev. Endocrinol..

[92] Singh H., Torralba M.G., Moncera K.J., DiLello L., Petrini J., Nelson K.E., Pieper R. (2019). Gastro-Intestinal and Oral Microbiome Signatures Associated with Healthy Aging. Geroscience.

[93] Wang J.J., Qie J.L., Zhu D.R., Zhang X.M., Zhang Q.Q., Xu Y.Y., Wang Y.P., Mi K., Pei Y., Liu Y. (2022). The Landscape in the Gut Microbiome of Long-Lived Families Reveals New Insights on Longevity and Aging—Relevant Neural and Immune Function. Gut Microbes.

[94] Li R.-D., Zheng W.-X., Zhang Q.-R., Song Y., Liao Y.-T., Shi F.-C., Wei X.-H., Zhou F., Zheng X.-H., Tan K.-Y. (2023). Longevity-Associated Core Gut Microbiota Mining and Effect of Mediated Probiotic Combinations on Aging Mice: Case Study of a Long-Lived Population in Guangxi, China. Nutrients.

[95] Kim B.-S., Choi C.W., Shin H., Jin S.-P., Bae J.-S., Han M., Seo E.Y., Chun J., Chung J.H. (2019). Comparison of the Gut Microbiota of Centenarians in Longevity Villages of South Korea with Those of Other Age Groups. J. Microbiol. Biotechnol..

[96] Rampelli S., Candela M., Turroni S., Biagi E., Collino S., Franceschi C., O’Toole P.W., Brigidi P. (2013). Functional Metagenomic Profiling of Intestinal Microbiome in Extreme Ageing. Aging.

[97] Kundu P., Lee H.U., Garcia-Perez I., Tay E.X.Y., Kim H., Faylon L.E., Martin K.A., Purbojati R., Drautz-Moses D.I., Ghosh S. (2019). Neurogenesis and Prolongevity Signaling in Young Germ-Free Mice Transplanted with the Gut Microbiota of Old Mice. Sci. Transl. Med..

[98] O’Toole P.W., Jeffery I.B. (2015). Gut Microbiota and Aging. Science.

[99] Hou Q., Huang J., Zhao L., Pan X., Liao C., Jiang Q., Lei J., Guo F., Cui J., Guo Y. (2023). Dietary Genistein Increases Microbiota-Derived Short Chain Fatty Acid Levels, Modulates Homeostasis of the Aging Gut, and Extends Healthspan and Lifespan. Pharmacol. Res..

[100] Tuikhar N., Keisam S., Labala R.K., Imrat, Ramakrishnan P., Arunkumar M.C., Ahmed G., Biagi E., Jeyaram K. (2019). Comparative Analysis of the Gut Microbiota in Centenarians and Young Adults Shows a Common Signature across Genotypically Non-Related Populations. Mech. Ageing Dev..

[101] Luo Y.-F., Cheng Z.-J., Wang Y.-F., Jiang X.-Y., Lei S.-F., Deng F.-Y., Ren W.-Y., Wu L.-F. (2024). Unraveling the Relationship between High-Sensitivity C-Reactive Protein and Frailty: Evidence from Longitudinal Cohort Study and Genetic Analysis. BMC Geriatr..

[102] Zhong W., Rao Z., Rao J., Han G., Wang P., Jiang T., Pan X., Zhou S., Zhou H., Wang X. (2020). Aging Aggravated Liver Ischemia and Reperfusion Injury by Promoting STING-Mediated NLRP3 Activation in Macrophages. Aging Cell.

[103] Liu Y., Wang T., Zhang F., Liu X., Hu Z., Zhang J., Chen H., Xiao J., You Q., Wu Z. (2025). Crosstalk between Liver Sinusoidal Endothelial Cells and Hepatocytes via IL-1α-IL1R1 Axis Exacerbates Ischaemia/Reperfusion Injury in Aged Livers. Gut.

[104] Xiao Y., Feng Y., Zhao J., Chen W., Lu W. (2025). Achieving Healthy Aging through Gut Microbiota-Directed Dietary Intervention: Focusing on Microbial Biomarkers and Host Mechanisms. J. Adv. Res..

[105] Chen S., Zhang Z., Liu S., Chen T., Lu Z., Zhao W., Mou X., Liu S. (2024). Consistent Signatures in the Human Gut Microbiome of Longevous Populations. Gut Microbes.

[106] Pang S., Chen X., Lu Z., Meng L., Huang Y., Yu X., Huang L., Ye P., Chen X., Liang J. (2023). Longevity of Centenarians Is Reflected by the Gut Microbiome with Youth-Associated Signatures. Nat. Aging.

[107] Yin J., Zhang Y., Liu X., Li W., Hu Y., Zhang B., Wang S. (2023). Gut Microbiota-Derived Indole Derivatives Alleviate Neurodegeneration in Aging through Activating GPR30/AMPK/SIRT1 Pathway. Mol. Nutr. Food Res..

[108] López-Otín C., Blasco M.A., Partridge L., Serrano M., Kroemer G. (2013). The Hallmarks of Aging. Cell.

[109] Saeedi B.J., Liu K.H., Owens J.A., Hunter-Chang S., Camacho M.C., Eboka R.U., Chandrasekharan B., Baker N.F., Darby T.M., Robinson B.S. (2020). Gut-Resident Lactobacilli Activate Hepatic Nrf2 and Protect against Oxidative Liver Injury. Cell Metab..

[110] Shang H.T., Huang C., Xiao Z.L., Yang P.C., Zhang S.Y., Hou X.H., Zhang L. (2023). Gut Microbiota-Derived Tryptophan Metabolites Alleviate Liver Injury via AhR/Nrf2 Activation in Pyrrolizidine Alkaloids-Induced Sinusoidal Obstruction Syndrome. Cell Biosci..

[111] Singh R., Chandrashekharappa S., Bodduluri S.R., Baby B.V., Hegde B., Kotla N.G., Hiwale A.A., Saiyed T., Patel P., Vijay-Kumar M. (2019). Enhancement of the Gut Barrier Integrity by a Microbial Metabolite through the Nrf2 Pathway. Nat. Commun..

[112] Zeng X.J., Li X.Q., Li X., Wei C., Shi C., Hu K.J., Kong D.L., Luo Q., Xu Y.L., Shan W. (2023). Fecal Microbiota Transplantation from Young Mice Rejuvenates Aged Hematopoietic Stem Cells by Suppressing Inflammation. Blood.

[113] Dawson P.A., Karpen S.J. (2015). Intestinal Transport and Metabolism of Bile Acids. J. Lipid Res..

[114] Foley M.H., O’Flaherty S., Barrangou R., Theriot C.M. (2019). Bile Salt Hydrolases: Gatekeepers of Bile Acid Metabolism and Host-Microbiome Crosstalk in the Gastrointestinal Tract. PLoS Pathog..

[115] Ganesan R., Gupta H., Jeong J.-J., Sharma S.P., Won S.-M., Oh K.-K., Yoon S.J., Han S.H., Yang Y.J., Baik G.H. (2024). Characteristics of Microbiome-Derived Metabolomics According to the Progression of Alcoholic Liver Disease. Hepatol. Int..

[116] Faerber V., Kuhn K., Garneata L., Kalantar-Zadeh K., Kalim S., Raj D., Westphal M. (2023). The Microbiome and Protein Carbamylation: Potential Targets for Protein-Restricted Diets Supplemented with Ketoanalogues in Predialysis Chronic Kidney Disease. Nutrients.

[117] Lai W., Zhang J., Sun J., Min T., Bai Y., He J., Cao H., Che Q., Guo J., Su Z. (2024). Oxidative Stress in Alcoholic Liver Disease, Focusing on Proteins, Nucleic Acids, and Lipids: A Review. Int. J. Biol. Macromol..

[118] Kawamoto S., Hara E. (2024). Crosstalk between Gut Microbiota and Cellular Senescence: A Vicious Cycle Leading to Aging Gut. Trends Cell Biol..

[119] Sun Y., Men Q., Ren X., Yan C., Song S., Ai C. (2024). Molecular Fucoidan Alleviated Alcohol-Induced Liver Injury in BALB/c Mice by Regulating the Gut Microbiota-Bile Acid-Liver Axis. Int. J. Biol. Macromol..

[120] Yang M., Zhang C.-Y. (2024). Interleukins in Liver Disease Treatment. World J. Hepatol..

[121] Zhao X., Yin F., Huang Y., Fu L., Ma Y., Ye L., Fan W., Gao W., Cai Y., Mou X. (2024). Oral Administration of Grape-Derived Nanovesicles for Protection against LPS/D-GalN-Induced Acute Liver Failure. Int. J. Pharm..

[122] Tian T., Xue Y., Song Z., Jin-Smith B., Barkin J., Ottallah M., Mannan M., Zhirkova A., Zhou D., Pi L. (2025). Targeted Clearance of Senescent Cells Alleviates Alcohol-Associated Liver Disease by Restoring Cellular Function and Immune Balance. Geroscience.

[123] Li S., Zhou C., Liu T., Zhang L., Liu S., Zhao Q., Liu J., Zhao W. (2024). Causal Relationships between the Gut Microbiota, Inflammatory Cytokines, and Alcoholic Liver Disease: A Mendelian Randomization Analysis. Front. Endocrinol..

